# Chlorpyrifos and other pesticide exposure and suspected developmental delay in children aged under 5 years: a case-control study in Phitsanulok, Thailand

**DOI:** 10.12688/f1000research.27874.2

**Published:** 2021-02-12

**Authors:** Yuwayong Juntarawijit, Uraiwan Chaichanawirote, Paphada Rakmeesri, Punaphop Chairattanasakda, Varintorn Pumyim, Chudchawal Juntarawijit

**Affiliations:** 1 Faculty of Nursing, Naresuan University, Phitsanulok, 65000, Thailand; 2 Faculty of Nursing, Kamphaeng Phet Rajabhat University, Kamphaeng Phet, 62000, Thailand; 3 Krabpung Health Promoting Hospital, Bangrakam District Health Office, Phitsanulok, 65140, Thailand; 4 Jomthong Health Promoting Hospital, Muang District Health Office, Phitsanulok, 65000, Thailand; 5 Faculty of Natural Resources and Environment, Naresuan University, Phitsanulok, 65000, Thailand

**Keywords:** Developmental disorder, child developmental delay, neurodevelopmental toxicity, pesticides neurotoxicity, chlorpyrifos

## Abstract

**Background**: Developmental delay among children under 5 years of age is a serious global public health problem and much research has been carried out to find potential causes. Pesticides - especially organophosphates - are suspected to be one of the main causes of the problem.  This study aimed to investigate the association between pesticide use by the mother during pregnancy and preschool children development using a case-control study.

**Methods**: Data on prenatal and postnatal pesticide exposure of 442 children with suspected developmental delay, and 413 controls with normal development were included for analysis. The children were matched for gender, age, and residency. Data on pesticide exposure were collected via interview with the mother, and data on pregnancy outcomes abstracted from hospital records.

**Results**: Chlorpyrifos exposure significantly increased the risk of developmental delay with an odds ratio (OR) of 3.71 (95% CI 1.03-13.36) for ever use of the pesticide, and an OR of 5.92 (95% CI 1.01-34.68) for postnatal exposure (p <0.05). Some other pesticides also had a positive association with developmental delay but none were statistically significant (p <0.05). Those pesticides were insecticide, fungicide, herbicide, and molluscicide. Individual pesticides with a positive association were glyphosate, paraquat, butachlor, methyl parathion (pholidon), savin, methomyl, endosulfan, carbosulfan, methamidophos, monochrotofos, mancozeb, and bordeaumixture.

**Conclusions**: This case-control study found that chlorpyrifos and some other pesticide exposure during pregnancy was positively associated with developmental delay in children aged under 5 years. Further research should be conducted to better understand this potential effect of pesticides on child neurodevelopment, and the public - especially those who plan to have families - should be informed.

## Abbreviations

ADHD, attention deficit and hyperactivity disorder; ASD, autism spectrum disorders; CPF, chlorpyrifos; DAP, dialkylphosphate metabolites; DD, developmental delay; IQ, intelligence quotient; OP, organophosphate; Ever, either prenatal exposure or postnatal exposure; PostN, postnatal exposure; PreN, prenatal exposure; SDD, suspected developmental delay.

## Introduction

Developmental delay in young children is a global public health concern. A study of 35 low- and middle-income countries reported that one in every three children below five years of age fails to reach their developmental potential ^1^. In Thailand, a national survey by the Ministry of Public Health reported that approximately 15% of children aged under 5 years are suspected to have a developmental delay (SDD) ^2^. In addition to stunting, inadequate cognitive stimulation, iodine and iron deficiency, malaria, intrauterine growth restrictions, maternal depression, exposure to violence ^3^, exposure to environmental toxicants including phthalates, bisphenol A, flame retardants, polycyclic aromatic hydrocarbon (PAHs), gas cooking ^4^, and heavy metals ^3^.

Pesticides of the acetylcholinesterase inhibitor group are another set of compounds suspected to affect neurodevelopment. In laboratory studies, this type of pesticide has been found to affect neuron cell and synaptic functions ^5^. Young children are also at a higher risk of pesticide effects because their bodies are not yet fully developed, and they also have a higher chance of exposure to environmental pesticides from engagement in high-risk behaviors, *e.g.* crawling on the floor, object-to-mouth behaviors, and playing with items found in the environment ^6^. A recent literature review indicated that 45 out of a total of 50 articles found a positive association between delayed neurodevelopment in young children and OP exposure ^7^. The neurological and behavioral developmental outcomes induced by pesticides include slower neonatal reflexes, delayed psychomotor and mental development ^8^, attention deficit ^9^, lower IQ ^10^, and autism spectrum disorder (ASD) ^11, 12^.

Regarding individual pesticides, chlorpyrifos (CPF) is the only OP pesticide that has been extensively studied, with most studies finding a positive association between exposure to CPF and child developmental delay. In a study among inter-city minority communities in New York City, researchers reported a positive association between levels of CPF in umbilical cord plasma and neurodevelopmental delay ^13^. Studies have also linked CPF exposure to poorer outcomes in working memory, visual motor coordination, color discrimination ^14^, and verbal comprehension ^15^, in addition to vision and hearing loss ^16^, and lower IQ ^17^. There is limited evidence that CPF exposure might also relate to ASD ^18^. On the other hand, a study of 2-year-old Mexican-American children found no association between cognitive function and CPF exposure ^19^, meaning this link is not yet conclusive.

Currently, in Thailand, the effects of CPF on child development have not been adequately studied. The objective of this case-control study was to analyze the association between suspected developmental delay (SDD) in children aged under 5 years living in Phitsanulok province, Thailand, and the use of CPF and other compounds during pregnancy. The results may be useful for SDD prevention, and for comparison to other similar studies in this field.

## Methods

### Study area

Phitsanulok province is in lower northern Thailand, located 370 km from Bangkok. It is a midsize province of 4,176 square miles, with nine districts, and a population of 866,891 people (density = 200 people per square mile). The capital city of the province is Muang district.

### Study participants

This study used a case-control design. Children diagnosed with suspected developmental delay (SDD) (cases) were compared with normal children (controls) with respect to pesticides exposure of the mother during pregnancy. Both cases and controls were children aged under 5 years who had participated in the National Child Developmental Screening Program. In Thailand, every child is screened for development progress at the ages of 9, 18, 30, and 42 months using the Developmental and Surveillance and Promotion Manual (DSPM) which modified from Denver Development Screening Test II (DDST-II). The screening is carried out by a trained nurse or health personnel at a health promoting hospital. In accordance with the DSPM manual, the children are evaluated in five skills, namely 1) gross motor skills), 2) fine motor skills, 3) receptive language skills, 4) expressive language skills, and 5) personal and social skills. If a child fails one or more of these skills, they are classified as having SDD. Children classified as having SDD were the target population in this study and were randomly selected to take part. The controls were children who attended the same hospital for the screening program but passed all five skills and were therefore classified as having normal development. The case and control groups were matched for gender, age, and area residence at assessment. Children with congenital anomalies or head trauma were excluded from the study.

### Sampling and sample size

Participants were children who participated in the screening program in selected local hospitals in the Bang Rakam and Muang districts of Phitsanulok province, Thailand. These two of the nine districts in the province were purposively selected to represent a rural area (Bang Rakam district) and an urban area (Muang district) of the province. A total of 15 out of 21 local hospitals in the Bang Rakam district, and 10 out of 30 hospitals in the Muang district were randomly chosen using a simple lottery method. The mothers of every child with SDD who met the inclusion criteria from the selected hospitals were invited to take part in the study at their appointment. For each case, a child with normal development was randomly selected from the hospital database matching for gender, age, and area residence.

The sample size was calculated to be 816 (408 cases and 408 controls) using OpenEpi online using the following assumptions: confident interval = 95%, power of detection = 80%, ratio of case to control = 1:1, proportion of control with exposure = 40, odds ratio = 1.5 ^20^.

### Questionnaire and data collection

Data on pesticide use and exposure during pregnancy was collected from the child’s mother using a constructed questionnaire (provided as Extended data in English) ^21^. Besides demographic data, the children’s mothers were asked “yes” or “no” questions concerning prenatal and postnatal use of pesticides. The exposure period was classified as “ever” for any prenatal and/or postnatal exposure to pesticides, “prenatal” for prenatal exposure to pesticides, and “postnatal” for postnatal exposure to pesticides. Pesticides were categorized into insecticides, herbicides, fungicides, rodenticides, and molluscicides. Exposure data for 14 individual compounds that are commonly used in Thailand and around the world were also collected. There were also questions for potential confounding factors such as occupation, monthly income, education, cigarette smoking, and alcohol use of the mother. Data on health status and pregnancy outcomes including delivery method, gestation, birth order, birth weight, and breast-feeding history, were retrieved from the hospitals’ medical records. Data collection took place in the participants’ homes, and was conducted between January and May, 2019. Data were collected by 60 village health volunteers who were trained to use the questionnaire and conduct the interviews.

The questionnaire was constructed by literature reviewed. The content validity of the questionnaire was tested by three experts in pediatric, obstetrics and gynecology and family medicine, and occupational health nursing. The index of Item Objective Congruence (IOC) was between 0.67–1.00. The questions were also tested for sequencing and understanding with a group of 30 women with similar characteristics of the intended participants.

### Data analysis

Demographic characteristics were analyzed with descriptive statistics and the results presented as frequency, percentage, mean, and standard deviation. Differences between groups were compared via t-test for continuous variables, and chi-square test for categorical data. The association between developmental delay and pesticide exposure was analyzed using multivariable logistic regression with odds ratios (OR) and a 95 percent confidence interval (CI) adjusted for mother age when pregnant (continuous), education (no school, primary school, secondary school, college degree), occupation (farmer, own business, civil servant, employee [formal], employee [general work], housewife, retired, unemployed), income (<5000 baht, 5000–9999, 10000–14999, 15000 or more), chronic disease (yes, no), alcohol consumption (yes, no), gestation (<37 weeks, 37 or more weeks), birth order (1, 2, 3 or more), delivery method (vaginal delivery, caesarean section, assisted delivery), baby weight (<2500 grams, 2500 grams or more), and breast-feeding (yes, no). Data was analyzed using IBM SPSS (version 26) software. Statistical significance was set at p <0.05 (2-tailed test).

### Ethical considerations

Ethical approval for this study was obtained from Naresuan University Institutional Review Board (Approval number 448/2019). Written informed consent to participate in the study and for attaining of their clinical details was obtained from the parents of the patients before data collection.

## Results

### Demographic data

From the dataset of 858 individuals, 855 records (413 cases, 442 controls) were used in the data analysis. Three records were not included for analysis because important information such as gender and age, was missing. Demographic data of the participants is shown in Table 1 and in the Underlying data ^22^. Most of the mothers were in the youngest age group with an average age of about 25 years. Most of them finished secondary school and had a monthly income of about 10,000 Thai Baht (300 USD) which is the minimum wage for Thailand. Only about 10% of them were farmers, and therefore reported using pesticides. There was a significantly higher proportion of mothers working as private employees, housewives, and civil servants. Most of the participants were healthy and had never drunk alcohol. One participant reported smoking cigarettes, and thus, was excluded from the data analysis. Data from the child’s medical records revealed that most of them, with the same proportion of case to control were born with spontaneous vaginal delivery (n=303, 69.5% and n=274, 67.5%, respectively). Compared to the control group, there were a higher proportion of cases born preterm and being the second or third child of the family. There was also a significant difference in birth weight and breastfeeding between groups. Although a higher percentage of cases had a birth weight of below 2,500 grams (n=64, 14.5% vs n=31, 7.6%, respectively), a lower percentage of them were not breast fed (n=25, 7.7% vs n=43, 12.8%, respectively).

**Table 1.** Demographic characteristics and some exposure factors of the study participants.

Characteristic	Control	Case	P-value
Child gender (n = 855)	n = 413	n = 442	0.916
Boy	222 (53.8)	236 (53.4)	
Girl	191 (46.2)	206 (46.6)	
Age of child at assessment, months (n = 855)	n = 413	n = 442	0.982
9	89 (21.5)	99 (22.4)	
18	112 (27.1)	121 (27.4)	
30	122 (29.5)	130 (29.4)	
42	90 (21.8)	92 (20.8)	
**Mother characteristic**			
Mother age when pregnant, years (n = 820)	n = 402	n = 418	0.358
≤20	106 (26.4)	98 (23.4)	
21–25	119 (29.6)	112 (26.8)	
26–30	82 (20.4)	101 (24.2)	
31–35	91 (22.6)	98 (23.4)	
≥36	4 (1.0)	9 (2.2)	
Mean ± SD	25.36 ± 6.51	26.17 ± 6.72	0.080
Range	13–42	13–46	
Education of mother (n = 847)	n = 407	n = 440	0.883
no school	10 (2.5)	14 (3.2)	
primary school	59 (14.5)	66 (15.0)	
secondary school	292 (71.7)	315 (71.6)	
college degree	46 (11.3)	45 (10.2)	
Occupation of mother (n = 845)	n = 407	n = 438	0.014 *
farmer	39 (9.6)	47 (10.7)	
own business	79 (19.4)	60 (13.7)	
civil servant	23 (5.7)	12 (2.7)	
Employee (formal)	59 (14.5)	77 (17.6)	
Employee (general work)	160 (39.3)	168 (38.4)	
housewife/ retired / unemployed	47 (11.5)	74 (16.9)	
Income of mother, baht (n = 813)	n = 394	n = 419	0.490
<5,000	84 (21.3)	93 (22.2)	
5,000–9,999	155 (39.3)	163 (38.9)	
10,000–14,999	74 (18.8)	64 (15.3)	
15,000 or more	81 (20.6)	99 (23.6)	
Mean ± SD	9854 ± 7352	10405 ± 8504	0.323
Cigarette smoking of mother (n = 856)	n = 414	n = 442	1.000
Never smoke	413 (99.8)	441 (99.8)	
Current smoker	1 (0.2)	1(0.2)	
Alcohol consumption of mother (n = 855)	n = 413	n = 442	0.039 *
Never drink	400 (96.9)	435 (98.4)	
Used to drink	6 (1.5)	0 (0)	
Currently drink	7 (1.7)	7 (1.6)	
Mother having chronic disease (n = 844)	n = 409	n = 435	0.197
No	383 (93.6)	397 (91.3)	
Yes	26 (6.4)	38 (8.7)	
**Child pregnancy/birth outcome**			
Child gestation period, week (n = 825)	n = 398	n = 427	0.599
37 or more	351 (88.2)	371 (86.9)	
<37	47 (11.8)	56 (13.1)	
Birth order (n = 847)	n = 410	n = 437	0.023 *
1	237 (57.8)	212 (48.5)	
2	125 (30.5)	158 (36.2)	
3 or more	48 (11.7)	67 (15.3)	
Delivery methods (n = 842)			0.288
vaginal delivery	274 (67.5)	303 (69.5)	
Caesarean section	126 (31.0)	131 (30.0)	
Assisted delivery	6 (1.5)	2 (0.5)	
Birth weight, gram (n = 850)	n = 410	n = 440	0.001 *
2,500 or more	379 (92.4)	376 (85.5)	
<2,500	31 (7.6)	64 (14.5)	
Ever breast-feeding (n = 662)	n = 336	n = 326	0.040 *
Yes	293 (87.2)	301 (92.3)	
No	43 (12.8)	25 (7.7)	
**Pesticide environmental exposure**			
Years of residence in the area (n = 850)	n = 412	n = 438	0.124
<5	77 (18.7)	97 (22.1)	
5–10	78 (18.9)	95 (21.7)	
11–20	84 (20.4)	63 (14.4)	
21–30	96 (23.3)	110 (25.1)	
31 or more	77 (18.7)	73 (16.7)	
Having family member working as a farmer (n = 830)	n = 399	n = 431	0.312
Yes	137 (34.3)	163 (37.8)	
No	262 (65.7)	268 (62.2)	
Distance from farm to home, km (n = 848)	n = 410	n = 438	0.280
<0.1	91 (22.2)	101 (23.1)	
0.1–0.5	111 (27.1)	97 (22.1)	
0.5–1.0	73 (17.8)	78 (17.8)	
2.0–5.0	76 (18.5)	104 (23.7)	
>5.0	59 (14.4)	58 (13.2)	
Frequency of farm enter (n = 855)	n = 413	n = 442	0.277
never	182 (44.1)	210 (47.5)	
<1 time per month	159 (38.5)	147 (33.3)	
>1 time per month	72 (17.4)	85 (19.2)	
Store pesticides in a house (n = 690)	n = 341	n = 349	1.00
Yes	50 (14.7)	51 (14.6)	
No	291 (85.3)	298 (85.4)	

Value expressed as number (percent) or mean ± standard error unless noted otherwise. * Statistically significant difference with p value <0.05.

### Environmental pesticide exposure

Roughly half of the participants had lived in the community for more than 10 years. With an equal proportion in the case and control groups, about 35% of participants had a family member working on a farm, 20% often entered farmland, and 14% stored pesticides in the house ( Table 1), yet around 70% lived within 1.0 km of farm land.

### Association of pesticide use and developmental delay

There were only 47 (10.4%) case mothers and 46 (11.4%) control mothers who reported ever using any pesticides during pregnancy. Table 2 presented odds ratio of SDD by types of pesticides the children were exposed to during pregnancy. Types of pesticides and exposure periods that were positively associated with SDD were insecticides (PostN), fungicides (PreN), fungicides (PostN), herbicides (PostN), and molluscicides (PostN). However, none of these ORs were statistically significant (p <0.05).

**Table 2.** Types of pesticides exposure of case and control and risk of suspected developmental delay.

Pesticide use	Control	Case	OR (crude)	OR (adjusted) **
Pesticide (Ever)				
No	366 (88.6)	395 (89.6)	1.0	1.0
Yes	47 (11.4)	46 (10.4)	0.91 (0.59-1.40)	0.97 (0.51-1.85)
Insecticide (Ever)				
No	373 (90.3)	403 (91.2)	1.0	1.0
Yes	40 (9.7)	39 (8.8)	0.90 (0.57-1.43)	0.97 (0.48-2.00)
Insecticide (PreN)				
No	375 (90.8)	410 (92.8)	1.0	1.0
Yes	38 (9.2)	32 (7.2)	0.77 (0.47-1.26)	0.84 (0.40-1.75)
Insecticide (PostN)				
No	397 (96.1)	421 (95.2)	1.0	1.0
Yes	16 (3.9)	21 (4.8)	1.24 (0.64-2.41)	1.61 (0.66-3.90)
Fungicide (Ever)				
No	389 (94.2)	412 (93.2)		
Yes	24 (5.8)	30 (6.8)	1.18 (0.68-2.06)	1.59 (0.71-3.56)
Fungicide (PreN)				
No	390 (94.4)	416 (94.3)	1.0	1.0
Yes	23 (5.6)	25 (5.7)	1.02 (0.57-1.83)	1.25 (0.54-2.91)
Fungicide (PostN)				
No	404 (97.8)	424 (96.1)	1.0	1.0
Yes	9 (2.2)	17 (3.9)	1.80 (0.79-4.08)	2.42 (0.82-7.14)
Herbicide (Ever)				
No	372 (90.1)	404 (91.4)	1.0	1.0
Yes	41 (9.9)	38 (8.6)	0.85 (0.54-1.36)	0.94 (0.48-1.86)
Herbicide (PreN)				
No	375 (90.8)	408 (92.3)	1.0	1.0
Yes	38 (9.2)	34 (7.7)	0.82 (0.51-1.33)	0.87 (0.43-1.73)
Herbicide (PostN)				
No	399 (96.6)	424 (95.9)	1.0	1.0
Yes	14 (3.4)	18 (4.1)	1.21 (0.59-2.47)	1.36 (0.53-3.47)
Rodenticide (Ever)				
No	397 (96.1)	425 (96.4)		
Yes	16 (3.9)	16 (3.6)	0.93 (0.46-1.89)	0.92 (0.36-2.35)
Rodenticide (PreN)				
No	397 (96.1)	427 (96.8)	1.0	1.0
Yes	16 (3.9)	14 (3.2)	0.81 (0.39-1.69)	0.81 (0.31-2.14)
Rodenticide (PostN)				
No	407 (98.5)	433 (98.2)	1.0	1.0
Yes	6 (1.5)	8 (1.8)	1.25 (0.43-3.64)	1.28 (0.34-4.86)
Molluscicide (Ever)				
No	392 (94.9)	420 (95.2)	1.0	1.0
Yes	21 (5.1)	21 (4.8)	0.93 (0.50-1.74)	0.92 (0.39-2.16)
Molluscicide (PreN)				
No	392 (94.9)	423 (95.9)	1.0	1.0
Yes	21 (5.1)	18 (4.1)	0.79 (0.42-1.51)	0.78 (0.32-1.87)
Molluscicide (PostN)				
No	404 (97.8)	429 (97.3)	1.0	1.0
Yes	9 (2.2)	12 (2.7)	1.26 (0.52-3.01)	2.11 (0.66-6.75)

Ever, either prenatal exposure or postnatal exposure; PreN, prenatal exposure; PostN, postnatal exposure. * Statistically significant with p value <0.05. **Adjusted for mother age when pregnant (continuous), education (no school, primary school, secondary school, college degree), occupation (farmer, own business, civil servant, employee [formal], employee [general work], housewife/ retired / unemployed, income (<5000 baht, 5000–9999, 10000–14999, 15000 or more), chronic disease (yes, no), alcohol consumption (yes, no), gestation (<37 weeks, 37 or more), birth order (1, 2, 3 or more), delivery method (vaginal delivery, caesarean section, assisted delivery), baby weight (<2500 grams, 2500 grams or more), and breast-feeding (yes, no).

Of 14 individual pesticides, exposure to CPF during pregnancy was significantly associated with child developmental delay. The associated odds ratio was significant for CPF (Ever) (OR = 3.71, 95% CI 1.03-13.36), and CPF (PostN) (OR = 5.92, 95% CI 1.01-34.68) ( Table 3). Risk of SDD were also increased with exposure to some other pesticides, including glyphosate(PostN), paraquat(PostN), butachlor(PostN), Methyl parathion/Pholidon(PostN), savin(PreN), savin (PostN), methomyl(PostN), endosulfan(PostN), carbosulfan(PostN), methamidophos(PreN), methamidophos(PostN), monochrotofos(PostN), mancozeb(PreN), mancozeb(PostN), bordeaumixture(PreN), and bordeaumixture(PostN); however, none were statistically significant.

**Table 3.** Individual pesticide exposure of case and control and risk of suspected developmental delay.

Pesticide use	Control	Case	OR (crude)	OR (adjusted) **
Glyphosate				
Glyphosate (Ever)				
No	377 (91.7)	409 (92.5)	1.0	1.0
Yes	34 (8.3)	33 (7.5)	0.90 (0.54-1.47)	0.93 (0.46-1.90)
Glyphosate (PreN)				
No	379 (92.2)	413 (93.4)	1.0	1.0
Yes	32 (7.8)	29 (6.6)	0.83 (0.49-1.40)	0.92 (0.45-1.91)
Glyphosate (PostN)				
No	400 (97.3)	426 (96.4)	1.0	1.0
Yes	11 (2.7)	16 (3.6)	1.37 (0.63-2.98)	1.32 (0.49-3.55)
Paraquat (Ever)				
No	381 (92.7)	417 (94.3)	1.0	1.0
Yes	30 (7.3)	25 (5.7)	0.76 (0.44-1.32)	0.88 (0.40-1.91)
Paraquat (PreN)				
No	383 (93.2)	419 (94.8)	1.0	1.0
Yes	28 (6.8)	23 (5.2)	0.75 (0.43-1.33)	0.85 (0.38-1.88)
Paraquat (PostN)				
No	403 (98.1)	431 (97.7)	1.0	1.0
Yes	8 (1.9)	10 (2.3)	1.17 (0.46-2.99)	1.63 (0.46-5.73)
Butachlor (Ever)				
No	400 (97.3)	435 (98.4)	1.0	1.0
Yes	11 (2.7)	7 (1.6)	0.59 (0.23-1.52)	0.88 (0.27-2.92)
Butachlor (PreN)				
No	400 (97.3)	437 (98.9)	1.0	1.0
Yes	11 (2.7)	5 (1.1)	0.42 (0.14-1.21)	0.58 (0.15-2.18)
Butachlor (PostN)				
No	407 (99.0)	436 (98.6)	1.0	1.0
Yes	4 (1.0)	6 (1.4)	1.40 (0.39-5.00)	2.85 (0.61-13.24)
Methyl parathion/ Pholidon (Ever)				
No	393 (95.4)	426 (96.8)	1.0	1.0
Yes	19 (4.6)	14 (3.2)	0.68 (0.34-1.37)	0.89 (0.32-2.48)
Methyl parathion/ Pholidon (PreN)				
No	394 (95.6)	427 (97.0)	1.0	1.0
Yes	18 (4.4)	13 (3.0)	0.67 (0.32-1.38)	0.95 (0.33-2.76)
Methyl parathion/ Pholidon (PostN)				
No	406 (98.5)	431 (98.0)	1.0	1.0
Yes	6 (1.5)	9 (2.0)	1.41 (0.50-4.01)	2.19 (0.57-8.40)
Savin (Ever)				
No	399 (97.3)	429 (97.5)	1.0	1.0
Yes	11 (2.7)	11 (2.5)	0.93 (0.40-2.17)	1.58 (0.50-4.96)
Savin (PreN)				
No	399 (97.3)	429 (97.5)	1.0	1.0
Yes	11 (2.7)	11 (2.5)	0.93 (0.40-2.17)	1.59 (0.51-5.00)
Savin (PostN)				
No	408 (99.3)	433 (98.4)	1.0	1.0
Yes	3 (0.7)	7 (1.6)	2.20 (0.57-8.56)	2.86 (0.62-13.27)
Chlorpyrifos (Ever)				
No	400 (97.3)	428 (97.1)	1.0	1.0
Yes	11 (2.7)	13 (2.9)	1.11 (0.49-2.49)	3.71 (1.03-13.36) *
Chlorpyrifos (PreN)				
No	401 (97.6)	430 (97.5)	1.0	1.0
Yes	10 (2.4)	11 (2.5)	1.03 (0.43-2.44)	2.97 (0.80-11.07)
Chlorpyrifos (PostN)				
No	406 (99.0)	433 (98.2)	1.0	1.0
Yes	4 (1.0)	8 (1.8)	1.88 (0.56-6.28)	5.92 (1.01-34.68) *
Methomyl (Ever)				
No	397 (96.6)	432 (98.0)	1.0	1.0
Yes	14 (3.4)	9 (2.0)	0.59 (0.25-1.38)	0.63 (0.22-1.80)
Methomy l(PreN)				
No	397 (96.6)	433 (98.2)	1.0	1.0
Yes	14 (3.4)	8 (1.8)	0.52 (0.22-1.26)	0.54 (0.18-1.61)
Methomyl (PostN)				
No	408 (99.3)	435 (98.6)	1.0	1.0
Yes	3 (0.7)	6 (1.4)	1.88 (0.47-7.55)	2.52 (0.52-12.23)
Endosulfan (Ever)				
No	394 (95.9)	431 (97.7)	1.0	1.0
Yes	17 (4.1)	10 (2.3)	0.54 (0.24-1.19)	0.65 (0.25-1.73)
Endosulfan (PreN)				
No	394 (95.9)	432 (98.0)	1.0	1.0
Yes	17 (4.1)	9 (2.0)	0.48 (0.21-1.10)	0.58 (0.21-1.56)
Endosulfan (PostN)				
No	408 (99.3)	434 (98.4)	1.0	1.0
Yes	3 (0.7)	7 (1.6)	2.19 (0.56-8.54)	5.16 (0.86-31.21)
Carbosulfan (Ever)				
No	401 (97.6)	434 (98.4)	1.0	1.0
Yes	10 (2.4)	7 (1.6)	0.65 (0.24-1.72)	0.78 (0.24-2.52)
Carbosulfan (PreN)				
No	401 (97.6)	436 (98.9)	1.0	1.0
Yes	10 (2.4)	5 (1.1)	0.46 (0.16-1.36)	0.51 (0.14-1.90)
Carbosulfan (PostN)				
No	409 (99.5)	435 (98.6)	1.0	1.0
Yes	2 (0.5)	6 (1.4)	2.82 (0.57-14.06)	4.47 (0.71-28.71)
Methamidophos (Tamaron) (Ever)				
No	107 (99.0)	437 (99.1)	1.0	1.0
Yes	4 (1.0)	4 (0.9)	0.93 (0.23-3.75)	1.98 (0.39-10.37)
Methamidophos (PreN)	407 (99.0)	437 (99.1)	1.0	1.0
No	4 (1.0)	4 (0.9)	0.93 (0.23-3.75)	1.99 (0.38-10.37)
Yes				
Methamidophos (PostN)	409 (99.5)	438 (99.3)	1.0	1.0
No	2 (0.5)	3 (0.7)	1.40 (0.23-8.43)	3.12 (0.42-23.38)
Yes				
Monochrotofos (Ever)				
No	406 (98.8)	438 (99.3)	1.0	1.0
Yes	5 (1.2)	3 (0.7)	0.56 (0.13-2.34)	1.63 (0.29-9.28)
Monochrotofos (PreN)				
No	406 (98.8)	438 (99.3)	1.0	1.0
Yes	5 (1.2)	3 (0.7)	0.56 (0.13-2.34)	1.63 (0.29-9.28)
Monochrotofos (PostN)				
No	408 (99.3)	438 (99.3)	1.0	1.0
Yes	3 (0.7)	3 (0.7)	0.93 (0.19-4.64)	3.12 (0.42-23.38)
DDT (Ever)				
No	397 (96.6)	434 (98.4)	1.0	1.0
Yes	14 (3.4)	7 (1.6)	0.46 (0.18-1.15)	0.44 (0.14-1.33)
DDT (PreN)				
No	398 (96.8)	434 (98.4)	1.0	1.0
Yes	13 (3.2)	7 (1.6)	0.49 (0.20-1.25)	0.53 (0.17-1.64)
DDT (PostN)				
No	408 (99.3)	438 (99.3)	1.0	1.0
Yes	3 (0.7)	3 (0.7)	0.93 (0.19-4.64)	1.42 (0.24-8.44)
Mancozeb (Ever)				
No	404 (98.3)	430 (97.5)	1.0	1.0
Yes	7 (1.7)	11 (2.5)	1.48 (0.57-3.85)	1.86 (0.58-5.89)
Mancozeb (PreN)				
No	404 (98.3)	430 (97.5)	1.0	1.0
Yes	7 (1.7)	11 (2.5)	1.48 (0.57-3.85)	1.86 (0.58-5.89)
Mancozeb (PostN)				
No	409 (99.5)	436 (98.9)	1.0	1.0
Yes	2 (0.5)	5 (1.1)	2.35 (0.45-12.16)	3.94 (0.59-26.19)
Bordeaumixture (Ever)				
No	409 (99.5)	436 (98.9)	1.0	1.0
Yes	2 (0.5)	5 (1.1)	2.35 (0.45-12.16)	4.00 (0.61-26.33)
Bordeaumixture (PreN)				
No	409 (99.5)	436 (98.9)	1.0	1.0
Yes	2 (0.5)	5 (1.1)	2.35 (0.45-12.16)	4.00 (0.61-26.33)
Bordeaumixture (PostN)				
No	409 (99.5)	438 (99.3)	1.0	1.0
Yes	2 (0.5)	3 (0.7)	1.40 (0.23-8.43)	3.12 (0.42-23.38)

Ever, either prenatal exposure or postnatal exposure; PreN, prenatal exposure; PostN, postnatal exposure. * Statistically significant with p value <0.05. **Adjusted for mother age when pregnant (continuous), education (no school, primary school, secondary school, college degree), occupation (farmer, own business, civil servant, employee [formal], employee [general work], housewife/ retired / unemployed, income (<5000 baht, 5000–9999, 10000–14999, 15000 or more), chronic disease (yes, no), alcohol consumption (yes, no), gestation (<37 weeks, 37 or more), birth order (1, 2, 3 or more), delivery method (vaginal delivery, caesarean section, assisted delivery), baby weight (<2500 grams, 2500 grams or more), and breast-feeding (yes, no).

## Discussion

The results showed CPF exposure during pregnancy and childhood SDD, with an odds ratio of 3.71 (95% CI 1.03-13.36) for ever using the pesticide (either prenatal or postnatal exposure), 2.97 (95% CI 0.80-11.07) for prenatal exposure, and 5.92 (95% CI 1.01-34.68) for postnatal exposure ( Table 3). Ever and postnatal exposure were found to be statistically significant. There was also a positive association, though not statistically significant, between SDD and other types of pesticides and individual compounds, including three herbicides [Glyphosate(PostN), Paraquat(PostN)), Butachlor(PostN)], two organophosphate insecticides [Pholidon (methyl parathion) (PostN), Tamaron (methamidophos)(PreN)], three carbamate insecticides [Savin(carbaryl), Methomyl(PostN), Carbosulfan(PostN)], and one organochlorine insecticide [Endosulfan(PostN)], and one fungicide [mancoceb(PreN)]. This is consistent with the literature: in an experimental study, CPF showed an ability to alter neuronal formation and structure in animal and human fetuses ^23, 24^. The synapse or neuronal junction, the site of transmission of nerve signals between two nerve cells, is perhaps a central target for neurodevelopmental susceptibility to pesticides. Since synapse plays a critical factor for the proper functioning of the neuro system, dysfunction of it, even subtle form, could lead to logic and psychiatric disorders, as well as subtler cognitive, psychomotor, and sensory defects ^5^. 

The results of epidemiological studies into SDD and pesticides have found a range of outcomes. In a study of Mexican American children aged 6–24 months, prenatal or child exposure to CPF was not associated with mental development, pervasive developmental disorder (a group of disorders characterized by delays in the development of socialization and communication skills), or behavioral problems ^19^. However, several other studies have found a positive association between prenatal exposure to CPF and neurodevelopmental problems. A cohort study of three-year-old children from minority communities in New York City, USA, reported a high exposure group (CPF levels of >6.17 pg/g in the mother’s plasma) to have a higher proportion of developmental delay, assessed by the psychomotor development index and the mental development index ^13^. A more recent study in Costa Rica found 6–9-year-old children with higher CPF exposure to have several neurobehavioral problems, including poorer working memory, visual motor coordination, and color discrimination, as well as parent-reported cognitive problems/inattention, oppositional disorder, and attention deficit hyperactivity disorder ^14^. A recent study of 9-month-old Thai infants reported an association between prenatal exposure to CPF and a reduction in grating visual acuity (OR = 0.64, 95% CI -1.22 to 0.06) ^16^.

One study has also linked prenatal exposure to CPF to lower IQ levels in children ^17^. Similarly, a study among children aged 5.9–11.2 years linked prenatal exposure to CPF to brain anomalies ^25^. This result has been replicated in a study of an adolescent group ^26^. Neurotoxic deficits have also been associated with CPF exposure in high-exposure occupations ^27^.

For groups of pesticides, most literature has focused on OPs due to their neurological toxic effects. These studies, usually using a cross-sectional or a cohort study design, have found positive correlations between OP metabolite in the mother’s urine and neurodevelopmental problems in the child ^7, 8^. Studies in the USA have reported prenatal exposure to OPs to increase the risk of abnormal reflexes in neonatal children (OR = 2.24, 95% CI 1.55-3.24) ^28^, and ADHD in male children at age five years (β = 1.3; 95% CI 0.4-2.1) ^9^. Living in close proximity to agricultural areas using OPs and other pesticides during pregnancy has also been related to ASD and developmental delay ^29^. A study in Taiwan using a case-control study design reported a dose-response relationship between OP metabolites in child urea and ADHD among children aged 4–15 years ^11^. Studies have also linked OP, carbamate, and pyrethroid pesticide exposure to lower IQ ^10, 15, 30^. A cohort study in Thailand also found lower motor and cognitive performance (using Bayley Scales of Infant and Toddler Development III [Bayley III]) among five-month old infants prenatally exposed to OP ^31^.

For other pesticides, data is limited. A study in Costa Rica reported a positive association of prenatal mancozeb exposure and lower social-emotional scores (β per 10-fold increase = -7.4 points [95% CI -15.2 to 0.4][measured by Bayley III]) in one-year-old infants ^32^. This is consistent with the present study which also found an elevated risk of SDD among those exposed to mancozeb, with odds ratio of 1.87 (95% CI 0.59-5.93) for prenatal exposure, and OR of 3.97 (95% CI 0.60-26.38) for postnatal exposure ( Table 3). A cohort study in Brittany, France, reported a negative association with neurocognitive development of 6-year-old children with prenatal exposure to pyrethroid ^33^. A cohort study in 4-year-old children in Greece reported the association between prenatal exposure to the organochlorine compounds and neurodevelopmental effects ^34^. A recent study in Indonesia reported a higher risk of small head circumference at birth to antenatal exposure to household non-OP pesticides (OR = -22.1 mm, 95% CI -36.5 to -7.6) ^35^.

Overall, the literature is limited and inconsistent regarding the critical duration of pesticide exposure developmental effects. In the current study, both prenatal and postnatal exposure was related to an increased risk of SDD ( Table 3), yet only postnatal exposure was significant. However, most of the previous studies on the prenatal and postnatal effects of CPF on neurodevelopmental, reported a positive effect only with prenatal exposure ^13, 16, 17, 25^. Unfortunately, only a limited number of studies have examined the effects of child neurodevelopment from both prenatal and postnatal exposure. One study that did ^36^ report a negative association of children’s developmental quotients (DQ) - a numerical indicator of a child’s growth to maturity across a range of psychosocial competencies - with prenatal exposure to OP but not with postnatal exposure. On the other hand, a cohort study in China found both prenatal and postnatal OP exposure increased the risk of developmental delay especially in the adaptive development (self-care skills) , among two-year old boys ^37^. In a laboratory study, CPF caused neurobehavioral impairment to a zebrafish when the exposure occurred in either the fertilization stage or embryonic stage ^38^.

In the current study, there were some limitations that need to be mentioned. First, there was a smaller number of participants who had used pesticides during pregnancy than expected, which limits the power of association between the variables. In addition, it is difficult to study the effect of low-level pesticide exposure on growth and development because the outcomes can be affected by several factors including biological factors ( *e.g.* stunting, infections, anemia, IUGR, preterm birth, birth weight, sex of the child, gestational age at delivery), psychosocial factors ( *e.g.* inadequate cognitive stimulation, exposure to violence, maternal depression, household dysfunction), and maternal sociodemographic factors ( *e.g.* poverty, low education, young age, smoking, drinking alcohol) ^39^. There may also have been a problem with recall bias during the interviews where participants may not have recalled or did not know the name of the pesticides they’d used in the past. However, if this did occur, it would have happened equally between groups. It was also very likely that study participants were exposed to pesticides in the environment, and this information bias could lower the strength of the reported association. 

## Conclusion

This case-control study found a negative association between chlorpyrifos and some other pesticide exposure during pregnancy and preschool child development. This effect was found in both prenatal and postnatal exposure. More research, using a larger sample size, is still needed to confirm the study results and to identify more individual pesticides which may impact prenatal and postnatal growth and development of children. This potential effect of pesticides on child neurodevelopment should receive more attention by researchers, and the public, especially those who plan to have families, should be informed.

## Data availability

### Underlying data

Figshare: child developmental delay and pesticide, Thailand. https://doi.org/10.6084/m9.figshare.13238501

This project contains the following underlying data:

- Child developmental delay and pesticide-database.csv (Collected demographic and child development data)
- Data dictionary-child development.docx (Word document containing dictionary for study dataset)

### Extended data

Figshare: Questionnaire-child developmental delay and pesticide, https://doi.org/10.6084/m9.figshare.13238507.v2

This project contains the following extended data:

- Questionnaire-child development and pesticide.docx (Study questionnaire in English)
- Questionnaire pesticide and development-Thai.docx (Study questionnaire in Thai)

Data are available under the terms of the Creative Commons Zero “No rights reserved” data waiver (CC0 1.0 Public domain dedication).
