# Supplementary material for: Chlorpyrifos and other pesticide exposure and suspected developmental delay in children aged under 5 years: a case-control study in Phitsanulok, Thailand
Source: F1000Res. 2021 Aug 24;9:1501. Originally published 2020 Dec 23. [Version 5] doi: 10.12688/f1000research.27874.5 (PMC8442115; doi:10.12688/f1000research.27874.5)
Supplement: Peer review history

# Peer review: Reviewer response for version 5

**DOI:** 10.5256/f1000research.76814.r92747

**Published:** 2021-09-14

**Authors:** Dana Boyd Barr

**Affiliations:** 1 Gangarosa Department of Environmental Health, Rollins School of Public Health, Emory University, Atlanta, GA, USA

I am happy with the revisions.

Is the work clearly and accurately presented and does it cite the current literature?

Partly

If applicable, is the statistical analysis and its interpretation appropriate?

Yes

Are all the source data underlying the results available to ensure full reproducibility?

Yes

Is the study design appropriate and is the work technically sound?

Partly

Are the conclusions drawn adequately supported by the results?

Partly

Are sufficient details of methods and analysis provided to allow replication by others?

Yes

Reviewer Expertise:

Biomonitoring, maternal child health, birth cohorts, exposure assessment, environmental epidemiology

I confirm that I have read this submission and believe that I have an appropriate level of expertise to confirm that it is of an acceptable scientific standard.

# Peer review: Reviewer response for version 4

**DOI:** 10.5256/f1000research.74905.r92022

**Published:** 2021-08-17

**Authors:** Dana Boyd Barr

**Affiliations:** 1 Gangarosa Department of Environmental Health, Rollins School of Public Health, Emory University, Atlanta, GA, USA

While I think the reviewers did do a good job of responding to my critiques, I don't feel they were adequately addressed in the paper.

 In the limitations section they state "However, if this did occur, it would have happened equally between groups. Exposure misclassification and self-selection bias were unlikely to occur because participants did not know or are aware of pesticides as a potential cause of SDD". This is simply untrue. One of the risks of retrospective exposure classification is that it is highly prone to misclassification. I think they need to acknowledge this in the limitations without dismissing it because they think it is non-differential bias. 

 Also, I think the lack of information on material diet and IQ could have affected the results. This should be stated in the limitations as well. Education is more of a determinant of wealth in the Thai populations I have evaluated and less related to IQ.

 Lastly, I think the manuscript does require some editing for English to ensure verb and noun agreement. Eg., 'data are' instead of 'data is' because the word 'data' is the plural of 'datum'. I noticed a few other occurrences of verb/noun disagreement as well.

Is the work clearly and accurately presented and does it cite the current literature?

Partly

If applicable, is the statistical analysis and its interpretation appropriate?

Yes

Are all the source data underlying the results available to ensure full reproducibility?

Yes

Is the study design appropriate and is the work technically sound?

Partly

Are the conclusions drawn adequately supported by the results?

Partly

Are sufficient details of methods and analysis provided to allow replication by others?

Yes

Reviewer Expertise:

Biomonitoring, maternal child health, birth cohorts, exposure assessment, environmental epidemiology

I confirm that I have read this submission and believe that I have an appropriate level of expertise to confirm that it is of an acceptable scientific standard, however I have significant reservations, as outlined above.

# Response

**Published:** 2021-08-20

**Authors:** Chudchawal Juntarawijit

**Affiliations:** Naresuan University, Thailand

**Comment:**

 While I think the reviewers did do a good job of responding to my critiques, I don't feel they were adequately addressed in the paper.

 In the limitations section they state "However, if this did occur, it would have happened equally between groups. Exposure misclassification and self-selection bias were unlikely to occur because the participants did not know or are aware of pesticides as a potential cause of SDD". This is simply untrue. One of the risks of retrospective exposure classification is that it is highly prone to misclassification. I think they need to acknowledge this in the limitations without dismissing it because they think it is non-differential bias. 

**Response**:

 Thank you very much again for your prompt response and valued comments and suggestions.

 Yes, we agree. Those statements have been deleted and some more information has been added to the limitations.

**Comment:**

 Also, I think the lack of information on material diet and IQ could have affected the results. This should be stated in the limitations as well. Education is more of a determinant of wealth in the Thai populations I have evaluated and less related to IQ.

**Response:**

 Yes, we agree. These two factors have been added along with related references.

**Comment:**

 Lastly, I think the manuscript does require some editing for English to ensure verb and noun agreement. Eg., 'data are' instead of 'data is' because the word 'data' is the plural of 'datum'. I noticed a few other occurrences of verb/noun disagreement as well.

**Response:**

 The English language has been further edited.

# Peer review: Reviewer response for version 3

**DOI:** 10.5256/f1000research.56696.r84051

**Published:** 2021-08-02

**Authors:** Dana Boyd Barr

**Affiliations:** 1 Gangarosa Department of Environmental Health, Rollins School of Public Health, Emory University, Atlanta, GA, USA

This manuscript describes the findings of a case-control study in Northern Thailand that evaluates suspected developmental delay (SDD) in relation to pesticide exposure as assessed through a questionnaire. While the study has several strengths such as the large size, matching cases and controls on sex, age, area of residence and hospital of SDD testing, and the standardized clinical testing of SDD, the study also has some pretty significant limitations/weaknesses that are not appropriately addressed.

1. No mention of maternal IQ was provided. Many studies have recognized that this is a potential predictor of child development.
2. Selection and recall bias: the authors state that "There may also have been a problem with recall bias during the interviews where participants may not have recalled or did not know the name of the pesticides they’d used in the past. However, if this did occur, it would have happened equally between groups. It was also very likely that study participants were exposed to pesticides in the environment, and this information bias could lower the strength of the reported association." but this is simply untrue. Mothers with children with SDD are far more likely to either recall precisely the chemicals that were used or to believe that the chemicals they used were the cause of SDD. Thus, I think this study is highly susceptible to recall bias. Similar issues with selection bias in that those that have more pronounced SDD were likely to volunteer or than those we less pronounced SDD or controls and the controls may have other uncontrolled differences.
3. Was information on crop provided by the mothers? This may have allowed the authors the opportunity to validate some of their questions.
4. The biggest limitation in this study is the retrospective exposure assessment via questionnaire. Ever recall using pesticides is likely quite imprecise and mothers may not wish to divulge that they were exposed to pesticides during pregnancy so as not to be "blamed" for their child's SDD. Exposure misclassification is not mentioned in the limitations yet all of the studies in the discussion including the one central Thai study used more objective measures of exposure (i.e., biological levels). When comparing the studies, the authors do not mention this. Similarly, the authors cannot derive any exposure magnitudes from this study which could obscure any underlying associations.
5. Because the authors findings differ so much from other studies that prospectively evaluated exposure and disease, they should elaborate more on why this is likely. For example, most prospective studies have found that prenatal exposures mostly drove any developmental delays while early childhood exposures had much less of an impact which is directly opposite of what the authors found. In all likelihood, the differences are likely because of exposure misclassification because the other studies had better exposure assessments ^1^^,^^2^^,^^3^^,^^4^^,^^5^.
6. It would be useful for the authors to let us know how their populations compare in demographic characteristics as the population in Northern Thailand or all of Thailand and how this may differ from other populations in the study. 

Is the work clearly and accurately presented and does it cite the current literature?

Partly

If applicable, is the statistical analysis and its interpretation appropriate?

Yes

Are all the source data underlying the results available to ensure full reproducibility?

Yes

Is the study design appropriate and is the work technically sound?

Partly

Are the conclusions drawn adequately supported by the results?

Partly

Are sufficient details of methods and analysis provided to allow replication by others?

Yes

Reviewer Expertise:

Pesticide exposure assessment, birth cohorts, maternal-child health, child neurodevelopment, north Thailand populations

I confirm that I have read this submission and believe that I have an appropriate level of expertise to state that I do not consider it to be of an acceptable scientific standard, for reasons outlined above.

## References

[1] : Prenatal Exposure to Organophosphorous Pesticides and Fetal Growth: Pooled Results from Four Longitudinal Birth Cohort Studies.*Environ Health Perspect*.124(7) : 10.1289/ehp.14093621084-9210.1289/ehp.140936226685281PMC4937849

[2] : Prenatal Organophosphorus Pesticide Exposure and Child Neurodevelopment at 24 Months: An Analysis of Four Birth Cohorts.*Environ Health Perspect*.124(6) : 10.1289/ehp.1409474822-3010.1289/ehp.140947426418669PMC4892910

[3] : Seven-year neurodevelopmental scores and prenatal exposure to chlorpyrifos, a common agricultural pesticide.*Environ Health Perspect*.2011;119(8) : 10.1289/ehp.10031601196-20110.1289/ehp.100316021507777PMC3237355

[4] : Prenatal exposure to organophosphate pesticides and IQ in 7-year-old children.*Environ Health Perspect*.2011;119(8) : 10.1289/ehp.10031851189-9510.1289/ehp.100318521507776PMC3237357

[5] : Organophosphate pesticide exposure and neurodevelopment in young Mexican-American children.*Environ Health Perspect*.2007;115(5) : 10.1289/ehp.9828792-810.1289/ehp.982817520070PMC1867968

# Response

**Published:** 2021-08-09

**Authors:** Chudchawal Juntarawijit

**Affiliations:** Naresuan University, Thailand

**Comments and responses**

**Comment:**

 This manuscript describes the findings of a case-control study in Northern Thailand that evaluates suspected developmental delay (SDD) in relation to pesticide exposure as assessed through a questionnaire. While the study has several strengths such as the large size, matching cases and controls on sex, age, area of residence and hospital of SDD testing, and the standardized clinical testing of SDD, the study also has some pretty significant limitations/weaknesses that are not appropriately addressed.

**Response:**

 Thank you very much for reviewing the manuscript and providing insightful comments. Yes, we agree that those issues are important and need to be clarified.

**Comment 1**: No mention of maternal IQ was provided. Many studies have recognized that this is a potential predictor of child development.

**Response**: Yes, maternal IQ might affect child development. Unfortunately, that data are not available. However, if we use the mothers’ education levels as the indicator, we found no difference among the case and control groups (Table 1).

**Comment 2:**

 Selection and recall bias: the authors state that "There may also have been a problem with recall bias during the interviews where participants may not have recalled or did not know the name of the pesticides they’d used in the past. However, if this did occur, it would have happened equally between groups. It was also very likely that study participants were exposed to pesticides in the environment, and this information bias could lower the strength of the reported association." but this is simply untrue. Mothers with children with SDD are far more likely to either recall precisely the chemicals that were used or to believe that the chemicals they used were the cause of SDD. Thus, I think this study is highly susceptible to recall bias. Similar issues with selection bias in that those that have more pronounced SDD were likely to volunteer or than those we less pronounced SDD or controls and the controls may have other uncontrolled differences.

**Response**:

 Concerning the problem of mothers with children with SDD are more likely to have a better recall of chemical use and be more willing to participate in the study. Yes, we agree that the problem will seriously affect the study result. However, that problem will occur only when the mothers are known, or aware that pesticide is a potential cause of SDD, which might be the case in Thailand. In the country, currently, data on toxic effects especially chronic consequences, such as developmental delay are limited and not publicized. One aim of this study was to explore the association and bring greater public attention to the potential effects of pesticide use in SDD cases. 

 In addition, from our data, we found only a slightly higher participation rate among the case than that of the control (86.67% vs. 80.98%).

 This information has been added to the result section.

**Comment 3:**

 Was information on crop provided by the mothers? This may have allowed the authors the opportunity to validate some of their questions.

**Response**:

 It is a good point. Unfortunately, we did not have that information. From what we know, most of the people in this area are rice farmers.

**Comment 4:**

 The biggest limitation in this study is the retrospective exposure assessment via questionnaire. Ever recall using pesticides is likely quite imprecise and mothers may not wish to divulge that they were exposed to pesticides during pregnancy so as not to be "blamed" for their child's SDD. Exposure misclassification is not mentioned in the limitations yet all of the studies in the discussion including the one central Thai study used more objective measures of exposure (i.e., biological levels). When comparing the studies, the authors do not mention this. Similarly, the authors cannot derive any exposure magnitudes from this study which could obscure any underlying associations.

**Response**:

 Yes, we agree that using exposure assessment via questionnaire might have some limitations. However, it may be the best tool for long-term exposure assessment, especially for a large survey study. Measurement of a biomarker in blood or urine is costly and represents only short-term exposure. This questionnaire technique was used in a well-recognized Agricultural Health Study in the United States [1] and many other previous studies.

 A few changes have been made and this information added to the discussion.

 The problem of fearing to be blamed for using pesticides during pregnancy may not occur in this study because, as we mentioned before, the mothers were not aware of pesticides as a potential cause of SDD. Therefore, we believed that exposure and misclassification might not be a big problem, and, if occurred, it would be equally distributed among the case and control groups.

 Yes, it is a good idea to explore the effect of exposure magnitudes when data are available. In this study, we try to collect data on the duration and intensity of pesticide use. However, since only a small number of mothers reported using pesticides during pregnancy, the data was too small for further analysis.

 Exposure misclassification has been added to the discussion.

**Comment 5:**

 Because the authors findings differ so much from other studies that prospectively evaluated exposure and disease, they should elaborate more on why this is likely. For example, most prospective studies have found that prenatal exposures mostly drove any developmental delays while early childhood exposures had much less of an impact which is directly opposite of what the authors found. In all likelihood, the differences are likely because of exposure misclassification because the other studies had better exposure assessments 1, 2, 3, 4, 5.

**Response**:

 Thank you for raising this important issue and sorry for causing any misunderstanding.

 First of all, we have to correct the point that our results are different from others. This is not true. Our results were consistent with the literature. In this study, we found that many pesticides especially chlorpyrifos was strongly associated with SDD. Concerning the window of periods of exposure, either prenatal or postnatal exposure to chlorpyrifos increased the risk of SDD. However, due to, maybe the small sample size, only the postnatal group showed a higher and more significant association. Currently, only a few studies reported data on both prenatal and postnatal exposure, further discussion is thus limited. By concept, both periods of exposure could affect SDD but greater effects are expected for prenatal exposure. 

 More information has been added to the discussion.

**Comment 6:**

 It would be useful for the authors to let us know how their populations compare in demographic characteristics as the population in Northern Thailand or all of Thailand and how this may differ from other populations in the study. 

**Response**:

 Sorry, we don’t have the data.

 However, based on previous studies, it was found that factors affecting children with SDD were the parents’ age, education level, occupation, income, and alcohol consumption [2]. However, in this study, the case was matched with controls by age and gender. In addition, those risk factors were included in the regression model to minimize the effect on the association.

**Reference**

1. Coble J, Thomas KW, Hines CJ, Hoppin JA, Dosemeci M, Curwin B, Lubin JH, Freeman LEB, Blair A, Sandler DP, Alavanja MCR. An Updated Algorithm for Estimation of Pesticide Exposure Intensity in the Agricultural Health Study. *International Journal of Environmental Research and Public Health*. 2011; 8(12):4608-4622. https://doi.org/10.3390/ijerph8124608
2. Kue-iad, N, Chairmay, B, and Woradet S. (2018). Early childhood development among Thai children aged under 5 years: a literature review. The Southern College Network Journal of Nursing and Public Health, 5(1): 281-296.

# Peer review: Reviewer response for version 2

**DOI:** 10.5256/f1000research.54363.r79509

**Published:** 2021-04-06

**Authors:** Ru-Lan Hsieh

**Affiliations:** 1 Department of Physical Medicine and Rehabilitation, Shin Kong Wu Ho-Su Memorial Hospital, Taipei Medical University, Taipei, Taiwan

I have some comments for this revised paper, as listed below:

1. It is surprising that the ages of mother ranged from 13-42 in controls and 13-46 in cases. Please provide the numbers of ages less than 18 in both groups, respectively, in Table 1.
2. Please provide the legal age for marriage in Thailand, and re-analyze the data by age less than 18 and >=18, in addition to the continuous data as analyzed.
3. The only significant factor to suspected developmental delay is Chlorpyrifos exposure in the present study. After the correction of crude OR in Table 3, the crude OR of ever Chlorpyrifos exposure is 1.11, prenatal exposure is 1.03, and postnatal exposure is 1.88. After adjustment, the OR of ever Chlorpyrifos exposure is 3.71 (p<0.05), prenatal exposure is 2.97, and postnatal exposure is 5.92 (p<0.05). The ORs after adjustment were much higher than the crude OR. Please provide the detailed regression model and correlation coefficient of each variable.
4. The authors did not adjust the factor of pesticide environment exposure for OR, why?
5. How could the authors get the contents of the pesticide as detailed as 14 kinds (such as Glyphosate, Paraquat, Butachlor, methyl parathion, Savin, Chlorpyrifos, etc.) just by questionnaires?
6. In the discussion section, the authors mentioned that *“This is consistent with the present study which also found an elevated risk of SDD among those exposed to mancozeb, with odds ratio of 1.87 for prenatal exposure, and OR of 3.97 for postnatal exposure (Table 3)”*. However, the OR did not reach statistically significant (p<0.05) in the present study. Therefore, the authors should not give this comment.

Is the work clearly and accurately presented and does it cite the current literature?

No

If applicable, is the statistical analysis and its interpretation appropriate?

No

Are all the source data underlying the results available to ensure full reproducibility?

Partly

Is the study design appropriate and is the work technically sound?

Partly

Are the conclusions drawn adequately supported by the results?

No

Are sufficient details of methods and analysis provided to allow replication by others?

Yes

Reviewer Expertise:

Rehabilitation medicine; pediatric rehabilitation; developmental delay

I confirm that I have read this submission and believe that I have an appropriate level of expertise to state that I do not consider it to be of an acceptable scientific standard, for reasons outlined above.

# Response

**Published:** 2021-05-05

**Authors:** Chudchawal Juntarawijit

**Affiliations:** Naresuan University, Thailand

*I have some comments for this revised paper, as listed below:*

*1. It is surprising that the ages of mother ranged from 13-42 in controls and 13-46 in cases. Please provide the numbers of ages less than 18 in both groups, respectively, in Table 1.*

**Response**:

 First, we would like to thank you for your time and efforts provide valuable comments and suggestions.

 As suggested, the numbers of participants aged less than 18 in each group have been added to Table 1. It can be noticed that the proportion of participants with <18 years in each group was similar to and there was no significant difference between the age group and developmental delay (p = 0.305).

*2. Please provide the legal age for marriage in Thailand, and re-analyze the data by age less than 18 and >=18, in addition to the continuous data as analyzed.*

**Response**:

 In Thailand, the law restricts marriage under the age of 17. In this study, we started with selected children with developmental delay and their controls. The information of the mother and pesticide exposure were collected later, without restriction on either the marital status or age of the mother.

 Further analysis using age as a categorical variable (<18, and 18 or more) was conducted as a suggestion, and the results, OR (adjusted2 in table 1-1). However, not much difference was observed as compared to the previous result when age was treated as a continuous variable (Table 3). It was also found that there was no literature to support a higher risk of developmental delay and among mothers below 18 years of age. Under the circumstances, therefore, it is better to treat age as a continuous variable.

*3. The only significant factor to suspected developmental delay is Chlorpyrifos exposure in the present study. After the correction of crude OR in Table 3, the crude OR of ever Chlorpyrifos exposure is 1.11, prenatal exposure is 1.03, and postnatal exposure is 1.88. After adjustment, the OR of ever Chlorpyrifos exposure is 3.71 (p<0.05), prenatal exposure is 2.97, and postnatal exposure is 5.92 (p<0.05). The ORs after adjustment were much higher than the crude OR. Please provide a detailed regression model and correlation coefficient of each variable.*

**Response**:

 The difference between crude and adjusted OR might be explained by the fact that there are several factors that can affect child development. The results support that control of confounding factors is important. In this study, the adjusted variables were: mother’s age when pregnant (continuous), education (no school, primary school, secondary school, college degree), occupation (farmer, own business, civil servant, employee [formal], employee [general work], housewife, retired, unemployed), income (<5000 baht, 5000–9999, 10000–14999, 15000 or more), chronic disease (yes, no), alcohol consumption (yes, no), gestation (<37 weeks, 37 or more weeks), birth order (1, 2, 3 or more), delivery method (vaginal delivery, cesarean section, assisted delivery), baby weight (<2500 grams, 2500 grams or more), and breastfeeding (yes, no). This information had been already presented in the Method section and in the table. In Table 3-1, the output of regression analysis of chlorpyrifos exposure and the correlation coefficient (B) of each variable was presented.

*4. The authors did not adjust the factor of pesticide environment exposure for OR, why?*

**Response**:

 This is a good point and thanks to you for the question.

 Yes, we did not adjust for pesticide environment exposure for several reasons. One reason is that the model has already contained many adjusted variables whereas the sample size was rather small with only 47 control and 46 cases reported using pesticides. It was also found that all of the environmental exposure variables were not significantly associated with developmental delay (Table 1). In addition, further analysis shows that these variables were strongly associated with pesticide use (data was not presented). For example, chlorpyrifos use was strongly associated with having a family member working as a farmer (p<0.001), year of residency in the area (p=0.010), frequency of farm entry (<0.001), and keeping pesticides in a house (p=0.009).

*5. How could the authors get the contents of the pesticide as detailed as 14 kinds (such as Glyphosate, Paraquat, Butachlor, methyl parathion, Savin, Chlorpyrifos, etc.) just by questionnaires?*

**Response**:

 Yes, the data on the 14 pesticides were collected using a face-to-face interview questionnaire. During the interview, village health volunteers asked each participant whether or not he/she ever used each individual pesticide, such as glyphosate, paraquat, butachlor, etc. This data collection method has been accepted and widely used in epidemiological studies. The method was suitable for a large study and studies that looked for the effects of long-term exposure because the blood or urine analyses are expensive and represent short-term exposure only. 

*6. In the discussion section, the authors mentioned that “This is consistent with the present study which also found an elevated risk of SDD among those exposed to mancozeb, with odds ratio of 1.87 for prenatal exposure, and OR of 3.97 for postnatal exposure (Table 3)”. However, the OR did not reach statistically significant (p<0.05) in the present study. Therefore, the authors should not give this comment.*

**Response**:

 Yes, it was true that the OR was not significant. However, when considered an association or effect, it should not depend solely on p-value and use it as a magic number. The problem of p-value and its misuse was discussed quite frequently in the literature. For example, Wasserstein, Schirm, and Lazar (2019) said that “Don’t believe that an association or effect is absent just because it was not statistically significant.”

 In the paper of Bonner *et al.* (2017) published in Environmental Health Perspective, many of the associations between pesticides and lung cancer were presented when the OR or HR did not reach statistically significant, p <0.05 or confident interval included 1. For example, “the association between pendimethalin use and lung cancer (HR = 1.50; 95% CI: 0.98, 2.31)”.

 Table 1-1 and Table 3-1 was in Figshare:

https://doi.org/10.6084/m9.figshare.14540154.v1

**Table 1-1**. Comparison of OR with two different adjustments of age, continuous (OR, adjusted) and categorical (OR, adjusted2) of some selected individual pesticides.

 Table 3-1. The output of regression analysis of chlorpyrifos exposure and the correlation coefficient (B) of each variable.

# Peer review: Reviewer response for version 2

**DOI:** 10.5256/f1000research.54363.r79508

**Published:** 2021-02-26

**Authors:** Zhijun Zhou

**Affiliations:** 1 School of Public Health, Key Laboratory of Public Health Safety of Ministry of Education, Fudan University, Shanghai, China

The author’s response to reviewer’s comments is acceptable, I support to accept this for indexing. Although, the limitation of this manuscript still exists; there is uncertainty of exposure assessment.

Is the work clearly and accurately presented and does it cite the current literature?

Yes

If applicable, is the statistical analysis and its interpretation appropriate?

Yes

Are all the source data underlying the results available to ensure full reproducibility?

Partly

Is the study design appropriate and is the work technically sound?

Partly

Are the conclusions drawn adequately supported by the results?

Partly

Are sufficient details of methods and analysis provided to allow replication by others?

Partly

Reviewer Expertise:

Children environmental health; toxicology

I confirm that I have read this submission and believe that I have an appropriate level of expertise to confirm that it is of an acceptable scientific standard.

# Peer review: Reviewer response for version 1

**DOI:** 10.5256/f1000research.30824.r76458

**Published:** 2021-01-19

**Authors:** Ru-Lan Hsieh

**Affiliations:** 1 Department of Physical Medicine and Rehabilitation, Shin Kong Wu Ho-Su Memorial Hospital, Taipei Medical University, Taipei, Taiwan

This study aimed to evaluate the association between pesticide use by mothers during pregnancy and preschool children development using a case-control design in Thailand. They concluded that chlorpyrifos exposure during pregnancy was positively associated with developmental delay in children less than 5 years. I have some comments as listed below:

  

1. The cases of children included in the present study were “suspected developmental delay” rather than “confirmed developmental delay”. Therefore, it would severely affect the results.
2. As the authors pointed out, there were only 47 (10.4%) of case mothers and 46 (11.4%) of control mothers reported ever using any pesticide during pregnancy. The case numbers were too small for comparison.
3. There were only 11 (2.7%) case mothers and 13 (2.9%) of control mothers had ever exposed to chlorpyrifos. Therefore, using these very small numbers of exposure to chlorpyrifos to evaluate the risk of developmental delay of children is not adequate at all. The results would severely mislead the readers.
4. The age ranges of mothers were between 13-42 and 13-46 in the control group and case group, respectively. Please re-analyze the mother’s age by below 18 (or 20) vs. above 18 (or 20).  
5. The only significant variable to developmental delay was the exposure to chlorpyrifos, and other variables were not statistically significantly associated with developmental delay. The authors calculated the crude odds ratios of chlorpyrifos ever exposure was 2.88, prenatal exposure was 2.34, and postnatal exposure was 4.18 as shown in Table 3. However, I found that it should be 1.10 in ever exposure, 1.02 in prenatal exposure, and 1.87 in postnatal exposure. Please recheck all variables’ odds ratios carefully.

Is the work clearly and accurately presented and does it cite the current literature?

No

If applicable, is the statistical analysis and its interpretation appropriate?

No

Are all the source data underlying the results available to ensure full reproducibility?

Partly

Is the study design appropriate and is the work technically sound?

Partly

Are the conclusions drawn adequately supported by the results?

No

Are sufficient details of methods and analysis provided to allow replication by others?

Yes

Reviewer Expertise:

Rehabilitation medicine; pediatric rehabilitation; developmental delay

I confirm that I have read this submission and believe that I have an appropriate level of expertise to state that I do not consider it to be of an acceptable scientific standard, for reasons outlined above.

# Response

**Published:** 2021-01-31

**Authors:** Chudchawal Juntarawijit

**Affiliations:** Naresuan University, Thailand

Reviewer II

 This study aimed to evaluate the association between pesticide use by mothers during pregnancy and preschool children development using a case-control design in Thailand. They concluded that chlorpyrifos exposure during pregnancy was positively associated with developmental delay in children less than 5 years. I have some comments as listed below:

 1. The cases of children included in the present study were “suspected developmental delay” rather than “confirmed developmental delay”. Therefore, it would severely affect the results.

**We agree with the reviewer that using confirm developmental delay may be the best outcome variable for studying conclusion. However, a number of children with developmental delay in the study area was so small, we have to rely on Suspected Developmental Delay (SDD). SDD is an important outcome variable and it has been used widely either in public health surveys for early identification of the prevalence rate, or to study risk factors. For example, a large study in 8 counties of rural China by Yang *et al.* (2019) ^1^ published in BMC Pediatrics, use SDD variables to evaluate the effects of care quality and development of children aged 1-59 months. Another study by Valla and team (2015) ^2^ also used SDD to study prevalence rates of developmental delay in Norwegian infants.**

 2. As the authors pointed out, there were only 47 (10.4%) of case mothers and 46 (11.4%) of control mothers reported ever using any pesticide during pregnancy. The case numbers were too small for comparison.

**Yes, we admitted that sample size is the study’s limitations. The number of mothers ever using pesticides is actually the exposure of interest. In this study, we collected data from about 800 participants, the minimum sample size required for this type of study, under the following assumption: 95% confident interval, power of detection = 80%, case to control = 1:1, odds ratio = 1.5, and control with exposure = 40% (as presented in the methods section). Unfortunately, only about ten percent of the mother had experience using pesticides. The problem was beyond our control, and with some other constraints, we have to report the result as such.**

**We did not completely agree that the sample size was ‘too small’. The issue is rather subjective, and it should depend mainly on the purpose of the study, and the statistics used for the analysis. The problem of using a small sample size is the lack of power of detection and precision. As seen in the study results, OR and other statistical parameters will not be significant. A small sample size will not completely destroy the usefulness of the study if it was analyzed with proper statistics and clearly presented.**

**According to the following article (DOI: 10.22004/ag.econ.103771** **), sample size should not be a main concern for logistic regression. Thus, we believed the result is good enough to be presented to research community.**

**As said by P. Mean. “A small sample size does not mean that your results are "wrong". It means that the data is consistent with a wide range of possible hypotheses.” ( http://www.pmean.com/11/WideInterval.html)**

 3. There were only 11 (2.7%) case mothers and 13 (2.9%) of control mothers had ever exposed to chlorpyrifos. Therefore, using these very small numbers of exposure to chlorpyrifos to evaluate the risk of developmental delay of children is not adequate at all. The results would severely mislead the readers.

**As mentioned before, the minimum sample size depends on the kind of statistic used for data analysis. If the sample size is too small, the OR obtained was not significant, as seen in many individual pesticides. This confirmed that the study results will not mislead the readers. We do the best to present study results. Data was analyzed with appropriate statistic, and the results were widely discussed. Therefore, readers can justify by themselves the reliability of the results.**

**It is not uncommon for studies to rely on small number of outcomes or exposure of interest, especially for rare diseases, e.g. developmental delay. For example, a study published in the Environmental Health Perspectives by Lui, *et al*. (2016), also included only 310 mother-infant pairs when studying the effects of organophosphate exposure and developmentally delayed. In this study, it reported OR between 9.75 (95% CI: 1.28, 73.98, p = 0.028) and 12.00 (95% CI: 1.23, 117.37, p = 0.033), notice a wide confidence interval. This conclusion came from the data nearly all with <10 number of cases in each group of exposure of interest (please see the manuscript and its supplemental materials https://doi.org/10.1289/EHP196).**

**The following are a list of some other studies that have a small sample size but yet provide useful information:**

- **Geetha, B., Sukumar, C., Dhivyadeepa, E. *et al.* Autism in India: a case–control study to understand the association between socio-economic and environmental risk factors. *Acta Neurol Belg* 119, 393–401 (2019). https://doi.org/10.1007/s13760-018-01057-4**
- **Rocha SGMO, Correia LL, Da Cunha AJLA, et al. Zika Virus Infection and Microcephaly: A Case-Control Study in Brazil. *Ann Glob Health*. 2019;85(1):116. Published 2019 Aug 28. doi: 10.5334/aogh.2394**
- **El-Baz F., Ismael, NA., and El-Din, SMN. (2011). Risk factors for autism: An Egyptian study. Egyptian Journal of Medical Human Genetics. 12(1). DOI: 10.1016/j.ejmhg.2011.02.011**

4. The age ranges of mothers were between 13-42 and 13-46 in the control group and case group, respectively. Please re-analyze the mother’s age by below 18 (or 20) vs. above 18 (or 20).

**Thank you for suggestions, it is a good point. At first, we considered the best age to have a healthy baby is between 25 and 35 years of age, and thus using 25 as a cut point. However, actually, there is no scientific data to support the idea. So, we agree and decide to recategorize the age group to be <20, 20-25, 26-30, 31-35, and ≥36 years, as suggested.**

 5. The only significant variable to developmental delay was the exposure to chlorpyrifos, and other variables were not statistically significantly associated with developmental delay. The authors calculated the crude odds ratios of chlorpyrifos ever exposure was 2.88, prenatal exposure was 2.34, and postnatal exposure was 4.18 as shown in Table 3. However, I found that it should be 1.10 in ever exposure, 1.02 in prenatal exposure, and 1.87 in postnatal exposure. Please recheck all variables’ odds ratios carefully.

**Thank you so much for your effort to identify the problem. All the data was checked and the error was found only with the crude OR. Data in Table 2 and Table 3 has been revised. Sorry for the mistake.**

**References:**

1. Yang, C., Liu, X., Yang, Y. *et al.* Quality of care and suspected developmental delay among children aged 1–59 months: a cross-sectional study in 8 counties of rural China. *BMC Pediatr* **19, **41 (2019). https://doi.org/10.1186/s12887-019-1406-x
2. Valla, L., Wentzel-Larsen, T., Hofoss, D. *et al.* Prevalence of suspected developmental delays in early infancy: results from a regional population-based longitudinal study. *BMC Pediatr* **15, **215 (2015). https://doi.org/10.1186/s12887-015-0528-z
3. de Moura DR, Costa JC, Santos IS, Barros AJD, Matijasevich A, Halpern R, Dumith S, Karam S, Barros FC. Risk factors for suspected developmental delay at age 2 years in a Brazilian birth cohort. *Paediatric and Perinatal Epidemiology* 2010; **24**: 211–221.

# Peer review: Reviewer response for version 1

**DOI:** 10.5256/f1000research.30824.r76367

**Published:** 2021-01-18

**Authors:** Zhijun Zhou

**Affiliations:** 1 School of Public Health, Key Laboratory of Public Health Safety of Ministry of Education, Fudan University, Shanghai, China

This manuscript reported the association between pesticide exposure of pregnant women and the suspected developmental delay of their children. It is interesting, but the quality of exposure assessment (description) is in question, only the questionnaire was used and there was obvious recall bias. How we can ensure the reality of pesticide exposure history? The authors should add more information on this point.

 Besides, several pesticide exposures were reported to have association with suspected developmental delay, what about the biological mechanism? Please add more literature on animal studies to support your results. Currently, only the similar studies were mentioned.

 Furthermore, the following minor comments should also be considered:

- The description that *“Three records were not included for analysis because important information such as gender and age, was missing”* in the section of results (Page 4) can’t be understood, since you have data of all cases firstly, then select the reference. 
- According to the description in the section of Sampling and sample size, all cases, including cases and controls, were from the database of children in the screening program. It is not case-control study, but cross-sectional study.
- About pesticide exposure history - is there data (records) of use of pesticides (types, amounts, use way, etc.) in these areas during the period when the mother were in pregnancy? It is important to use such data to confirm the answer of these mothers, specifically the use stage (pre-N, or post-N).

Is the work clearly and accurately presented and does it cite the current literature?

Yes

If applicable, is the statistical analysis and its interpretation appropriate?

Yes

Are all the source data underlying the results available to ensure full reproducibility?

Partly

Is the study design appropriate and is the work technically sound?

Partly

Are the conclusions drawn adequately supported by the results?

Partly

Are sufficient details of methods and analysis provided to allow replication by others?

Partly

Reviewer Expertise:

Children environmental health; toxicology

I confirm that I have read this submission and believe that I have an appropriate level of expertise to confirm that it is of an acceptable scientific standard, however I have significant reservations, as outlined above.

# Response

**Published:** 2021-01-31

**Authors:** Chudchawal Juntarawijit

**Affiliations:** Naresuan University, Thailand

Reviewer I

 1. This manuscript reported the association between pesticide exposure of pregnant women and the suspected developmental delay of their children. It is interesting, but the quality of exposure assessment (description) is in question, only the questionnaire was used and there was obvious recall bias. How we can ensure the reality of pesticide exposure history? The authors should add more information on this point.

**The risk of health effects associated with long-term exposure to pesticides is difficult to assess in epidemiologic studies. Direct measurement of exposure is often not feasible in large studies. Also, measurement of a biomarker in blood or urine is costly and represent a short-term exposure. For long-term exposure, using a questionnaire collecting data on duration and intensity of pesticide use might be more appropriate. This practice was found in a large study like Agricultural Health Study in the United State ^1^.**

**It may inappropriate to discuss issue in the paper.**

 Besides, several pesticide exposures were reported to have association with suspected developmental delay, what about the biological mechanism? Please add more literature on animal studies to support your results. Currently, only the similar studies were mentioned.

**Thanks for reminding the point. More information on biological mechanism has been added.**

 2. The description that *“Three records were not included for analysis because important information such as gender and age, was missing”*  in the section of results (Page 4) can’t be understood, since you have data of all cases firstly, then select the reference. 

**The information was missing during data entry. The problems occur with only a few cases thus it should not significantly affect the result.**

 3. According to the description in the section of Sampling and sample size, all cases, including cases and controls, were from the database of children in the screening program. It is not case-control study, but cross-sectional study. 

**Yes, all cases were from the same database. However, the study designed is a case-control study because case and control groups were selected based on their disease status (developmental delay). Then, pesticide exposure data in the past of the two groups were collected. If it was a cross-sectional study, all children should have been randomly selected, regardless of their developmental status, and the data on either diseases or exposure should have been collected simultaneously.**

 4. About pesticide exposure history - is there data (records) of use of pesticides (types, amounts, use way, etc.) in these areas during the period when the mother were in pregnancy? It is important to use such data to confirm the answer of these mothers, specifically the use stage (pre-N, or post-N).

**We agree that the data will be useful. Unfortunately, there was no such data in the area, especially data of individual pesticides. As we mentioned before, using a questionnaire may be the best and the only way to collect data on long-term historical exposure of pesticides.**

**References:**

 [1] Coble J, Thomas KW, Hines CJ, Hoppin JA, Dosemeci M, Curwin B, Lubin JH, Freeman LEB, Blair A, Sandler DP, Alavanja MCR. An Updated Algorithm for Estimation of Pesticide Exposure Intensity in the Agricultural Health Study. *International Journal of Environmental Research and Public Health*. 2011; 8(12):4608-4622. https://doi.org/10.3390/ijerph8124608
